# Dissociation between Corneal and Cardiometabolic Changes in Response to a Time-Restricted Feeding of a High Fat Diet

**DOI:** 10.3390/nu14010139

**Published:** 2021-12-29

**Authors:** Prince K. Akowuah, Aubrey Hargrave, Rolando E. Rumbaut, Alan R. Burns

**Affiliations:** 1 College of Optometry, University of Houston, Houston, TX 77204, USA; aubreych@standford.edu (A.H.); arburns2@central.uh.edu (A.R.B.); 2 Children’s Nutrition Research Center, Baylor College of Medicine, Houston, TX 77030, USA; rrumbaut@bcm.edu; 3 Center for Translational Research on Inflammatory Diseases (CTRID), Michael E. DeBakey Veterans Affairs Medical Center, Houston, TX 77030, USA

**Keywords:** corneal dysregulation, high fat diet, obesity, neutrophils, platelets, time-restricted feeding

## Abstract

Mice fed a high fat diet (HFD) ab libitum show corneal dysregulation, as evidenced by decreased sensitivity and impaired wound healing. Time-restricted (TR) feeding can effectively mitigate the cardiometabolic effects of an HFD. To determine if TR feeding attenuates HFD-induced corneal dysregulation, this study evaluated 6-week-old C57BL/6 mice fed an ad libitum normal diet (ND), an ad libitum HFD, or a time-restricted (TR) HFD for 10 days. Corneal sensitivity was measured using a Cochet-Bonnet aesthesiometer. A corneal epithelial abrasion wound was created, and wound closure was monitored for 30 h. Neutrophil and platelet recruitment were assessed by immunofluorescence microscopy. TR HFD fed mice gained less weight (*p* < 0.0001), had less visceral fat (*p* = 0.015), and had reduced numbers of adipose tissue macrophages and T cells (*p* < 0.05) compared to ad libitum HFD fed mice. Corneal sensitivity was reduced in ad libitum HFD and TR HFD fed mice compared to ad libitum ND fed mice (*p* < 0.0001). Following epithelial abrasion, corneal wound closure was delayed (~6 h), and neutrophil and platelet recruitment was dysregulated similarly in ad libitum and TR HFD fed mice. TR HFD feeding appears to mitigate adipose tissue inflammation and adiposity, while the cornea remains sensitive to the pathologic effects of HFD feeding.

## 1. Introduction

Obesity can precipitate a cascade of systemic conditions, including metabolic syndrome, cardiovascular disease, non-alcoholic liver disease, and type 2 diabetes [1,2,3]. Hence, the increasing global prevalence of obesity is very concerning. The World Health Organization considers a person to be overweight when their body mass index (BMI) is >25 and obese when their BMI is >30. Globally, about 2 billion individuals are overweight or obese, accounting for approximately 30% of the world population [4]. In the United States, almost half (42.4%) of the adult population is obese [5].

In addition to the cardiometabolic complications of obesity, there is emerging interest in its effects on vision. Recent studies show obesity is associated with an early loss of corneal nerve density and function [6,7,8]. Loss of corneal nerve structure and function is detrimental to the health of the cornea, as corneal nerves release neurotrophic factors, which play a central role in the maintenance of corneal epithelial integrity and corneal transparency [9,10]. The pathologic effects of obesity on the cornea can appear quickly and before the onset of hyperglycemia [6,7,8]. In mice fed an obesogenic high fat diet (HFD) for 10 days, not only is there a loss of corneal nerve sensitivity, but there is also a noticeable delay in corneal wound healing following a central epithelial abrasion [6]. Normal and efficient corneal wound healing is crucial for the preservation of corneal transparency, and when compromised, the cornea becomes susceptible to infection, ulceration, and opacification [11,12]. Hence, there is a need to find novel strategies for preventing the corneal nerve degeneration and impaired corneal wound healing observed in diet-induced obesity.

The energy imbalance resulting from increased consumption of energy-dense high-fat, -sugar, and -salt diets, collectively referred to as the Western diet, has been the main driving force of the current obesity epidemic [13,14,15]. The temporal distribution of food intake plays an important role in mediating the metabolic and health outcomes of a given diet. Chrono-nutrition, the coordination of food intake with the daily rhythm of an organism, offers a promising approach to forestalling or treating the cardiometabolic effects of diets [16,17]. The range of dietary strategies that manipulate the timing of food consumption are collectively known as intermittent fasting. Intermittent fasting strategies can be broadly grouped into alternate-day fasting (ADF), whole-day fasting, and time-restricted (TR) feeding [18]. ADF typically involves alternating periods of 36 h of fasting followed by 12 h of ad libitum food consumption [18,19]. Some forms of ADF allow one meal containing ~25% of the individual’s baseline caloric needs, consumed typically in the afternoon during fast periods [20]. Whole-day fasting strategies usually involve 1–2 days of severe caloric restriction or complete food abstinence followed by ad libitum feeding the rest of the week [21,22]. TR feeding is a form of chrono-nutrition, in which food intake is restricted to a period (usually 6–10 h/day) during the active hours of an organism. Evidence from animal studies [23,24,25] shows TR feeding elicits favorable metabolic effects, including protection against weight gain, hyperinsulinemia, insulin resistance, and adipose inflammation in response to an HFD, without requiring alterations in caloric intake or nutrient composition [23,24,25,26]. Even though TR feeding mitigates the cardiometabolic complications of diets, its utility in preventing the corneal dysregulation observed with ad libitum HFD feeding remains unknown. In this study, we used a diet-induced obesity mouse model to compare corneal and cardiometabolic changes in response to a TR feeding regimen. We evaluated the effect of TR feeding on the corneal nerve function loss and impaired corneal wound healing observed in ad libitum HFD fed mice. We hypothesized that TR feeding would mitigate the corneal nerve function loss and impaired corneal wound healing observed in ad libitum HFD feeding.

## 2. Materials and Methods

### 2.1. Mice

**Figure 1:**
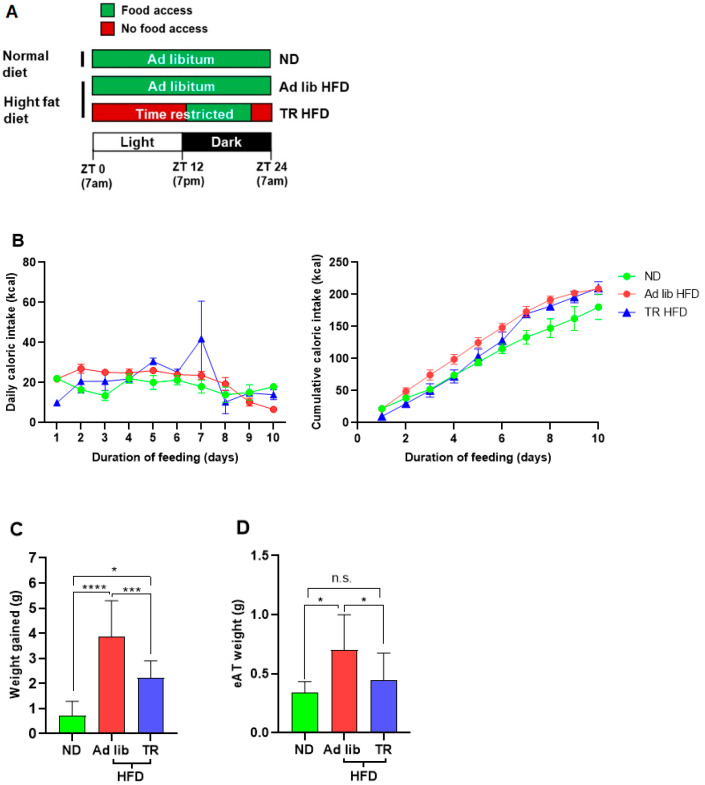
Time-restricted feeding attenuated body weight gain and adiposity. (**A**) Schematic representation of the different feeding regimens. TR HFD fed mice were allowed access to food from 8 p.m. (ZT13) through 4 a.m. (ZT21). (**B**) Caloric intake in ND, ad libitum HFD, and TR HFD fed mice. Left: average daily caloric intake. Right: Cumulative average caloric intake over the 10-day experimental feeding period. (**C**) Body weight gain and (**D**) visceral adiposity (eAT) in ND, ad libitum HFD, and TR HFD fed mice. ND—normal diet; TR—time-restricted; HFD—high fat diet; n.s.—not significant; * *p* ≤ 0.05; *** *p* ≤ 0.001; **** *p* ≤ 0.0001.

Six-week-old C57BL/6 male mice (Jackson Laboratory) were housed in the University of Houston (UH) and Baylor College of Medicine (BCM) Children’s Nutrition Research Center vivaria under temperature control and a 12:12 light-dark cycle environment (two mice per cage). Mice housed at UH were divided into three 10-day feeding groups. Power analysis revealed that a sample size of ≥6 mice per group would have 80% statistical power to reliably detect an effect size of 0.5 (50%) in diet-induced corneal changes, assuming a 0.05 significance level. The first group (*n* = 6) was fed a normal chow diet (ND; 5V5R, LabDiet, St. Louis, MO, USA) ad libitum. The second group (*n* = 8) was fed an HFD (Diet #112734; Dyets Inc., Bethlehem, PA, USA) ad libitum, while the third group (*n* = 8) was fed the HFD using a TR regimen. Two groups of mice were housed at BCM (TR HFD, *n* = 10, and TR HFD, *n* = 10), and these mice were used to evaluate body composition adipose tissue inflammation only (see Section 2.3 below). The nutritional composition of the diets is summarized in Table 1. Figure 1A shows a schematic representation of the experimental feeding regimens. For TR feeding, mice were allowed access to food for 8 h (between 8 p.m. and 4 a.m.). A TR ND group was not included in the current study because previous studies [23,25,27] found no significant differences in cardiometabolic parameters between ad libitum ND and TR ND groups. For experiments at Baylor College of Medicine Children’s Nutrition Research Center, food access was monitored using an automated metabolic chamber. For experiments at the University of Houston, food access was monitored by manually switching the mice from a cage with food and water to a cage with just water. Mice fed on the ad libitum regimens were also transferred between cages at the same time.

### 2.2. Ethics Statement

The study was approved by the Institutional Animal Care and Use Committee (IACUC) at Baylor College of Medicine (protocol #: AN-2721) and at the University of Houston (protocol #: 16-005). All procedures were performed according to the Association for Research in Vision and Ophthalmology (ARVO) Statement for the Use of Animals in Ophthalmic and Vision Research.

### 2.3. Body Composition and Adipose Tissue Inflammation

Body composition studies were performed in the Mouse Metabolic Research Unit at the USDA/ARS Children’s Nutrition Research Center, Baylor College of Medicine. All mice were weighed at the start and end of experimental feeding. Prior to euthanasia, body composition was determined at the end of the experimental feeding using quantitative magnetic resonance (qMR) imaging. After euthanasia by carbon dioxide inhalation followed by cervical dislocation, epididymal adipose tissue (eAT) was harvested, weighed, and then processed for flow cytometric analysis to evaluate adipose inflammation. Antibodies against the following markers were used: CD45 and CD3 (BD Biosciences, San Diego, CA, USA) and F4/80 (eBioscience, San Diego, CA, USA). CD45^+^/CD3^+^ were identified as T cells, and CD45^+^/F4/80^+^ cells were identified as macrophages.

### 2.4. Corneal Nerve Function

A Cochet-Bonnet aesthesiometer (Richmond Products, Albuquerque, NM, USA) was used to measure the sensitivity of the cornea to tactile stimulation. The Cochet-Bonnet aesthesiometer has a thin nylon filament, which was held perpendicular to the central cornea. Starting at the maximum filament length (6.0 cm), the length was systematically decreased (0.5 cm increments) until a blink was observed when the filament was pressed against the central corneal surface. Shortening the filament length stiffens the filament, resulting in greater pressure. A decrease in corneal sensitivity is indicated when increased filament pressure is needed to elicit a blink.

### 2.5. Corneal Wound Healing

Mice were anesthetized via intraperitoneal injection of ketamine/xylazine (80 mg/8 mg/kg body weight). Using a trephine and a blunt golf spud, a 2 mm central corneal epithelial abrasion wound was created on the left eye. Wounding was performed in the morning of each day (between 8 a.m.–12 p.m.) to avoid confounding circadian effects on wound healing and the inflammatory response. The size of the wound opening was imaged at the time of wounding (0 h) and 12, 18, 24, and 30 h after wounding using sodium fluorescein staining. Briefly, under isoflurane anesthesia, 1μL of 1% sodium fluorescein was pipetted onto the corneal surface. The wound was then imaged using a stereomicroscope equipped with a digital camera and blue light illumination. The wound area, denoted by the pooled fluorescein in the image, was then measured with ImageJ software (NIH, Bethesda, MD, USA). The results for each time point were expressed as a percentage of the original wound size.

### 2.6. Immunofluorescence Staining

Mice were euthanized at 30 h after wounding, a timepoint known to correlate with peak neutrophil infiltration into the central cornea during wound healing [28]. The eyes were enucleated and fixed in phosphate buffered saline (PBS) containing 2% paraformaldehyde (Tousimus Research Corporation, Rockville, MD, USA) for 45 min at room temperature. Corneas were then excised from the eyeball, permeabilized in PBS containing 2% bovine serum albumin (BSA) and 0.01% TritonX-100 for 15 min, followed by blocking in PBS containing 2% BSA for an additional 45 min at room temperature. Corneas were then incubated overnight at 4 °C in a cocktail of fluorescently-labeled antibodies (5–10 µg/mL). The following antibodies were used: anti-CD31 antibody (for limbal blood vessels) (BioLegend, San Diego, CA, USA), anti-Ly6G antibody (for neutrophils) (BD Pharmingen, San Diego, CA, USA), and anti-CD41 antibody (for platelets) (BioLegend, San Diego, CA, USA). DAPI (4′,6-diamidino-2-phenylindole, Sigma-Aldrich, St. Louis, MO, USA) was added to the cocktail to visualize nuclei and mitotic figures. Corneas were flat mounted on a microscope slide in Airvol (Celanese, Dallas, TX, USA) and imaged with a DeltaVision epifluorescence light microscope (GE Life Sciences, Pittsburg, PA, USA). Full-thickness images were captured with a 30× silicon lens with an image size of 381 µm × 381 µm and a z-section thickness step size of 0.5 microns.

### 2.7. Morphometric Analysis of Neutrophil and Platelet Recruitment

To assess neutrophil infiltration, images were taken in the central, paracentral, parawound, paralimbal, and limbal regions of the cornea in each quadrant, as previously reported [28,29]. Both DAPI and Ly-6G staining were used to identify extravascular neutrophils. Neutrophil counts in each region from the 4 petals, except the center (which had counts from only one field per cornea), were averaged and expressed as neutrophils per field. For extravascular platelet assessment, the entire corneal limbus was imaged in each petal, and platelet counts from the four petals were summed together. Extravascular platelet counts were then expressed as platelets/mm^2^ of limbal area, since extravascular platelets are non-motile and remain within the limbus [30]. Epithelial cell division was assessed by counting mitotic figures, which were visualized via DAPI staining.

### 2.8. Statistical Analysis

Data were analyzed using GraphPad Prism 6 (GraphPad Software, La Jolla, CA, USA). Mean ± standard deviation was used to summarize data. Unpaired *t*-tests and ANOVAs (one-way, two-way, and repeated measures with Tukey post hoc tests for multiple comparisons) were used to analyze data when appropriate. For all statistical analyses, an alpha level of ≤0.05 was considered significant.

## 3. Results

### 3.1. Time-Restricted Feeding Attenuated Weight Gain, Adiposity, and Adipose Inflammation

Figure 1A shows the feeding regimen for each mouse group. Despite similar caloric intakes (Figure 1B), mice fed the TR HFD gained less weight compared to mice fed the ad libitum HFD (*p* < 0.0001, Figure 1C). In addition, TR HFD fed mice had less eAT mass compared to the ad libitum HFD (*p* = 0.015, Figure 1D). Although TR HFD fed mice gained more weight than ND mice (*p* = 0.013), the two groups of mice did not differ significantly in eAT weight (*p* = 0.685). TR HFD feeding resulted in significantly lower body fat mass compared to ad libitum HFD feeding, as determined by qMR imaging (*p* = 0.0003, Table 2).

HFD feeding induces inflammation in adipose tissue, which involves the infiltration of macrophages into adipose tissue [31,32]. Adipose tissue macrophages (CD45^+^/F4/80^+^) were six-fold lower in TR HFD fed mice compared to ad libitum HFD fed mice (*p* = 0.032, Table 2). T cells play active roles in diet-induced adipose tissue inflammation, increasing early in adipose tissue and likely preceding the infiltration of macrophages [33,34,35]. In this study, TR HFD fed mice had nine-fold fewer T cells (CD45^+^/CD3^+^) in adipose tissue compared to ad libitum HFD fed mice (*p* = 0.028, Table 2). Furthermore, the total number of leukocytes (CD45^+^) in adipose tissue was eight-fold lower in TR HF fed mice compared to ad libitum HFD fed mice (*p* = 0.018, Table 2).

### 3.2. Time-Restricted Feeding Did Not Prevent Dysregulation of Corneal Homeostasis

**Figure 2:**
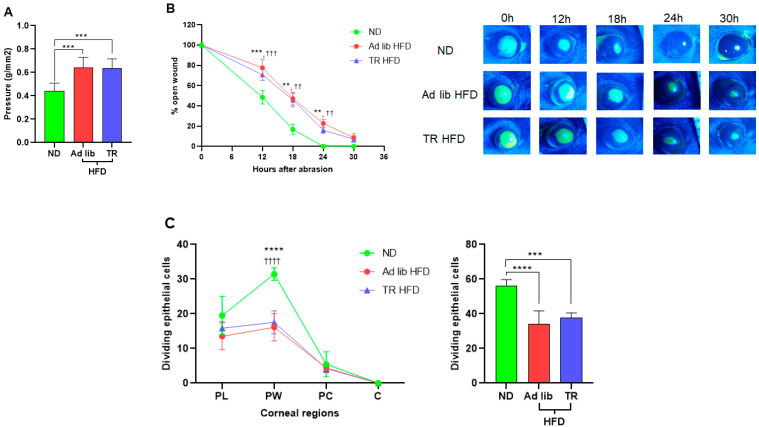
Time-restricted feeding did not attenuate reduced corneal sensitivity and impaired corneal wound healing. (**A**) Corneal sensitivity in 10 day ND fed mice compared to ad libitum and TR HFD fed mice (*n* ≥ 6 per group). (**B**) Corneal wound closure in mice fed an ad libitum ND, an ad libitum HFD and a TR HFD. Left: Wound closure kinetics indicating complete wound closure by 24 h in ad libitum ND fed mice, while the wound remained open up to 30 h in the ad libitum HFD and TR HFD fed mice. Right: Representative images of the epithelial wound immediately after wounding, 12 h, 18 h, 24 h and 30 h after wounding. (**C**) Left: Dividing basal epithelial cells in each region of the cornea. Right: Sum of dividing basal epithelial cells in four fields of view from the paralimbus to the center of the cornea at 30 h after wounding (*n* ≥ 6 per group). Data expressed as means ± SD. ** *p* ≤ 0.01, *** *p* ≤ 0.001, **** *p* ≤ 0.0001 (ad libitum HFD compared to ad libitum ND); †† *p* ≤ 0.01, ††† *p* ≤ 0.001, †††† *p* ≤ 0.0001 (TR HFD compared to ND).

As expected, ad libitum HFD feeding caused a significant reduction in corneal nerve sensitivity (Figure 2A), as the filament pressure required to elicit a blink was increased when compared to ND mice. TR HFD feeding did not prevent this reduction, and corneal sensitivity was similar to that in ad libitum HFD mice (Figure 2A). Closure of a 2 mm epithelial abrasion wound in C57BL/6 mice is usually complete by 24 h after wounding [6]. As expected, at 24 h after wounding, wound closure was complete in mice fed the ND. However, in mice fed the ad libitum HFD, wound closure was delayed by ~6 h, and this delay was also observed in TR HFD fed mice (Figure 2B). A two-fold reduction in basal epithelial cell division at the parawound was also noted for mice fed the ad libitum HFD or TR HFD (Figure 2C).

**Figure 3:**
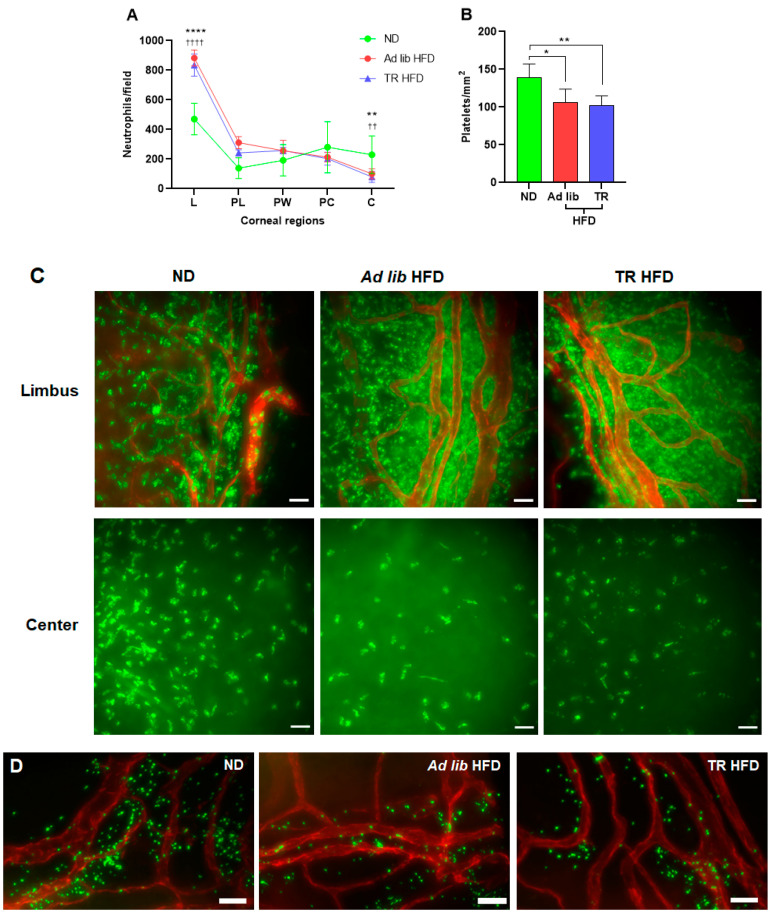
Inflammatory response of the cornea to abrasion. (**A**) The distribution of neutrophils over the limbal (L), paralimbal (PL), parawound (PW), paracentral (PC) and central (C) regions of the corneal stroma at 30 h after wounding (*n* ≥ 6). (**B**) Data showing platelet counts at the limbus 30 h after wounding (*n* ≥ 6). (**C**) Representative images of the abraded cornea of ad libitum ND, ad libitum HFD, and TR HFD 30 h after abrasion, showing extravasated neutrophils (green, anti-Ly-6G) and vasculature (red, anti-CD31) at the limbus. (**D**) Representative images of the abraded cornea of ad libitum ND, ad libitum HFD and TR HFD 30 h after abrasion, showing extravasated platelets (green, anti-CD41) and vasculature at the limbus. Data expressed as mean ± SD. * *p* ≤ 0.05, ** *p* ≤ 0.01, **** *p* ≤ 0.0001 (ad libitum HFD compared to ad libitum ND); †† *p* ≤ 0.01 and †††† *p* ≤ 0.0001 (TR HFD compared to ad libitum ND). Scale bars: C = 34 µm; D = 35 µm.

Neutrophil extravasation at the limbus and subsequent migration to the center of the cornea has been shown to be necessary for efficient corneal wound healing [28]. Thirty hours post-wounding, peripheral limbal images from ad libitum HFD and TR HFD fed mice had twice as many neutrophils (Figure 3A,C) and 25% fewer platelets (Figure 3B,D) than ad libitum ND fed mice. Conversely, the accumulation of neutrophils at the wound center was reduced in the ad libitum HFD and TR HFD groups (Figure 3A).

## 4. Discussion

The aim of the current study was to compare corneal and cardiometabolic changes in response to a TR HFD feeding regimen. We found that (1) although mice on the TR feeding consumed an equivalent number of calories as those on the ad libitum HFD feeding, TR feeding attenuated body weight gain, adiposity, and adipose tissue inflammation; (2) despite the effects of TR HFD feeding on mitigating systemic dysregulation, it had no effect on the reduced corneal sensitivity, impaired corneal wound healing, or dysregulated neutrophil and platelet infiltration observed in ad libitum HFD fed mice.

Given the clinical and economic burden associated with obesity [36,37], it has become imperative to find preventive and interventional strategies for obesity. With the pivotal role of lifestyle choices, such as nutrition and activity level, in the rising obesity epidemic, lifestyle modification has been the go-to strategy for preventing/treating obesity. This strategy has the advantage of being low-cost and easy to access as compared to surgical or pharmacological strategies for treating obesity [38]. The temporal distribution of caloric intake has been shown to be a significant contributor to the cardiometabolic effects of diet. Under ad libitum feeding conditions, HFD is known to blunt diurnal feeding rhythms, shortening an organism’s fasting period while prolonging the feeding period [25]. This disturbs various metabolic pathways entrained by the feed-fast cycle, predisposing the organism to obesity and other metabolic diseases. TR feeding, a form of chrono-nutrition that de-emphasizes reduction in caloric intake, is considered to be a potential behavioral strategy for preventing obesity [23,24,25,26]. In humans, there are two main types of TR feeding: early TR feeding, where caloric intake is restricted to early (morning) or middle (afternoon) of the day and late TR feeding, where caloric intake is restricted to late (evening) in the day. These two TR feeding strategies produce diverging results. Early TR feeding reduces body weight gain, insulin levels, and systemic inflammation and increases insulin sensitivity [39,40,41], while late TR feeding worsens or has little effect on these cardiometabolic parameters [42,43,44]. This divergence may be explained by the circadian system. The circadian system produces ~24 h rhythms in behavior, physiology, and metabolism through feedback loops involving the transcription and translation of genes, collectively known as clock genes (e.g., *Bmal1, Clock, Per1/2, Cry 1/2*) [45,46,47]. This leads to oscillations in the expression and level of downstream target molecules. In humans, for instance, the expression and activity of key metabolic hormones such as insulin and cortisol exhibit a rhythm, with peak expression and activity in the morning and nadir in the evening. Insulin sensitivity also exhibits a 24 h rhythm, with a peak and a nadir in the morning and evening, respectively, suggesting the morning is an optimal time for food intake [48]. The opposite is observed in nocturnal animals such as mice and rats.

In the current study, ad libitum HFD feeding resulted in significant adiposity (body weight gain and eAT deposition) relative to the ad libitum ND feeding. Daily TR HFD feeding (8 h) attenuated adiposity and diet-induced adipose inflammation without altering nutritional/caloric intake. This is in agreement with reports by other investigators [24,25,26,49]. Hatori et al. [25] reported that despite equivalent caloric consumption between ad libitum and TR access to HFD, TR feeding in mice protected against obesity, hyperinsulinemia, hepatic steatosis and systemic inflammation. The beneficial effects of TR eating have also been reported in humans. Wilkinson et al. [50] reported improvement in cardiometabolic health (body weight, blood pressure, and atherogenic lipids) of individuals with metabolic syndrome following a two-week regimen of 10 h TR eating. Jamshed et al. [51] reported improvement in glucose levels, lipid metabolism, and circadian clock gene expression in overweight individuals following a four day 6 h early TR eating schedule. Jones et al. [52] also reported that 8 h early TR eating for two weeks improved whole body insulin sensitivity and skeletal muscle glucose and branched-chain amino acid (BCAA) uptake. An important difference between these TR eating studies in humans and the current mouse study is that the human studies employed TR eating as an interventional strategy, while the current study employed TR feeding as a preventative strategy. Although TR feeding of the HFD resulted in significant reduction in body weight gained compared to the ad libitum HFD, mice on the TR HFD gained more weight than ad libitum ND. However, visceral adiposity (eAT) was not different between TR HFD and ad libitum ND groups. A possible reason for this discrepancy may be the fact that several fat deposits (e.g., epididymal, retroperitoneal, mesenteric, inguinal, cervical, etc.) contribute to the overall body weight. A limitation of our study is that only epididymal fat deposits were used to assess visceral adiposity. Hence, TR HFD and ad libitum ND groups may have differed in the weight of other fat deposits, which in turn may have contributed to the observed difference in overall body weight.

An important determinant of the overall metabolic signal needed to maintain body weight at a steady-state value is the duration of fasting. Insulin is an anabolic hormone that facilitates fatty acid synthesis and storage, alongside its effects on glucose uptake and storage. Diet-induced obesity is associated with insulin hypersecretion and insulin resistance [53,54]. TR feeding is largely centered on the prolonged daily fasting period (14–16 h), which gives the body a chance to repair oxidative damage, leading to metabolic adaptations that sustain weight loss [55,56]. Fasting leads to decreased insulin production and reduced levels of insulin in the circulation [57,58,59] and also increases fatty acid utilization [60]. Fasting also forces a shift in metabolic pathway usage from a glucose-driven oxidative phosphorylation to ketone- and fatty acid-dependent metabolism [61,62]. Ketones are produced from fatty acids by the liver through a process known as ketogenesis, and fasting maintains ketogenesis [63]. The shift in fuel utilization from glucose to ketones reinforces the metabolic circadian rhythm while reducing oxidative stress and systemic inflammation [63,64,65]. The gut microbiome has been shown to be instrumental in metabolism and the metabolic effects of diets [66,67]. In humans, the two main phyla of the gut microbiome are Firmicutes (F) and Bacteroidetes (B), and an increase in the F/B ratio has been linked to obesity and increased metabolic disorders [68]. TR feeding exerts beneficial effects on the gut microbiome by decreasing the F/B ratio [69]. Hence, the observed reduction in adiposity in mice fed a TR HFD to levels comparable to those found in mice fed the ad libitum ND may be due to a combination of the effects fasting has on insulin signaling, fuel utilization, and the gut microbiome.

Our previous study [6] shows ad libitum HFD feeding in mice for 10 days reduces corneal sensitivity, and in response to a central epithelial abrasion, corneal wound closure is delayed. Efficient corneal epithelial wound closure depends on a carefully regulated inflammatory response. This response includes a carefully orchestrated recruitment of neutrophils and platelets, which provide essential mediators (e.g., VEGF) that support epithelial cell division and nerve regeneration. This inflammatory response is dysregulated within 10 days of ad libitum feeding [6]. In the current study, we confirm these findings for ad libitum HFD feeding and now report an identical corneal pathology for mice fed the TR HFD. With respect to corneal wound healing, the dysregulation of the inflammatory response was indistinguishable in ad libitum HFD and TR HFD fed mice. Extravascular platelet counts at the limbus were reduced, and there was a marked reduction in neutrophil migration toward the wound center, which likely explains the excessive accumulation at the limbus. Hence, while TR HFD feeding is clearly able to mitigate adipose inflammation (increased numbers of T cells and macrophages; see Table 2) seen in ad libitum HFD fed mice, it does not prevent the HFD-induced dysregulation of the inflammatory response seen after corneal abrasion. The similar and dysregulated inflammatory response seen in wounded corneas of ad libitum HFD and TR HFD fed mice likely accounts for the similarly reduced rates of epithelial division and wound closure seen under both feeding regimens.

Most TR feeding studies in humans and rodents have focused on alterations in adiposity and metabolic health. Although the preventive utility of TR feeding in mitigating cardiometabolic effects of diet-induced obesity has been studied extensively [24,25,26,49], its effects on nerve health and wound healing are less well studied. Some animal studies suggest that TR feeding may benefit nerve health by delaying or protecting against the onset of neurodegenerative diseases. Kentish et al. [70] reported that TR feeding restores ad libitum HFD-induced loss of gastric vagal afferent mechanosensitivity. Two studies demonstrated that TR feeding in a mouse model of Huntington’s disease improves autonomic nervous function and motor coordination [71,72]. An association between TR eating and cognitive status has also been reported in humans. In a cross-sectional cohort study, individuals adherent to TR eating (10 h eating window restriction) were found to be less likely to show cognitive impairment compared to those on an ad libitum eating schedule [73]. The benefits of TR feeding on the nervous system are believed to act through the brain-derived neurotrophic factor (BDNF). TR feeding increases BDNF levels [51]. BDNF is a neurotrophin that is crucial for the development, maintenance, and plasticity of the nervous system. TR feeding increases ketone production due to the extended fasting [74]. Increased ketone levels in cortical and hippocampal neurons, specifically beta-hydroxybutyrate, induce transcription of BDNF [75]. Our current study suggests TR feeding does little to protect corneal nerve health, as the effects of the HFD continue to decrease corneal nerve sensitivity despite the TR regimen. Hence, the beneficial effects of TR feeding may be nerve- and tissue-specific.

TR feeding is largely centered on the prolonged daily fasting periods (14–16 h), which give the body a chance to repair oxidative damage [55,56]. A possible explanation for the lack of benefit of TR feeding on corneal dysregulation may be found in the effect of extended fasting on the expression of insulin-like growth factors (IGFs) and the importance of IGFs to corneal homeostasis and wound healing. IGF-I has been shown to promote corneal epithelial cell migration and cell proliferation, processes that are important for corneal wound healing [76,77]. Following epithelial abrasion, the secretion of IGF-I and IGF-II increases in the corneal epithelium, and expression of their native receptor, IGF-1R, also increases in the limbal epithelium [78,79]. Fasting significantly reduces circulating levels of IGFs and gene transcription of IGF-I [80]. Importantly, in the skin, the decrease in IGF-1 expression caused by extended fasting has been linked to impaired wound healing [81,82]. Future studies are warranted to investigate the effect of fasting/TR HFD feeding on the expression of IGFs and their corresponding receptors in the cornea and if exogenous IGFs can enhance corneal homeostasis and wound healing in HFD fed mice.

The dissociation between the corneal response and systemic adipose response to dietary strategies is not observed with TR feeding alone. We have previously reported such a dissociation in the response to a diet-reversal strategy [6]. Even though switching from an HFD to an ND mitigated weight gain and visceral adiposity, the HFD feeding induced a heightened inflammatory state of the cornea, which persisted after diet-reversal. Thus, it is evident from the current TR feeding study and our prior diet-reversal study that the cornea is especially vulnerable to the effects of an HFD, even when diet-based therapeutic strategies designed to mitigate adipose inflammation and adiposity are employed.

## 5. Conclusions

In summary, a “short-term” consumption of an ad libitum HFD causes corneal dysregulation in the form of corneal sensitivity loss and impaired corneal wound healing. While TR feeding attenuates systemic parameters such as adiposity and adipose tissue inflammation, it does not attenuate or prevent the corneal dysregulation observed in ad libitum HFD feeding. This suggests that corneal changes are dissociated from the systemic changes regulated by TR feeding.

## Figures and Tables

**Table 1:** Nutritional composition of the ND and HFD used in the study.

	ND (%KCal)	HFD (%KCal)
Fat	14.8	42
Sugar	0	30
Complex carbohydrate	62.1	12.8
Protein	23.1	15.2

ND—normal diet; HFD—high fat diet.

**Table 2:** Body composition and adipose tissue (eAT) immune cells of HFD fed mice.

	Ad Libitum HFD	TR HFD
Fat mass (g)	4.99 ± 0.79	3.56 ± 0.62 ***
Lean mass (g)	22.13 ± 1.02	23.16 ± 1.41
CD45^+^	47,894 ± 36,355	5720 ± 4520 *
CD45^+^/F4/80^+^	25,413 ± 20,865	3926 ± 3569 *
CD45^+^/CD3^+^	19,629 ± 16,557	2181 ± 2020 *

Data are mean ± SD. Abbreviations: TR—time restricted; HFD—high fat diet. * *p* < 0.5; *** *p* < 0.001.

## Data Availability

The data presented in this study are available on request from the corresponding author.
